# Determination of EGFR Endocytosis Kinetic by Auto-Regulatory Association of PLD1 with μ2

**DOI:** 10.1371/journal.pone.0007090

**Published:** 2009-09-18

**Authors:** Jun Sung Lee, Il Shin Kim, Jung Hwan Kim, Wonhwa Cho, Pann-Ghill Suh, Sung Ho Ryu

**Affiliations:** 1 Division of Molecular and Life Sciences, Pohang University of Science and Technology, Pohang, Kyungbuk, Republic of Korea; 2 Department of Chemistry, University of Illinois at Chicago, Chicago, Illinois, United States of America; Thomas Jefferson University, Kimmel Cancer Center, United States of America

## Abstract

**Background:**

Upon ligand binding, cell surface signaling receptors are internalized through a process tightly regulated by endocytic proteins and adaptor protein 2 (AP2) to orchestrate them. Although the molecular identities and roles of endocytic proteins are becoming clearer, it is still unclear what determines the receptor endocytosis kinetics which is mainly regulated by the accumulation of endocytic apparatus to the activated receptors.

**Methodology/Principal Findings:**

Here we employed the kinetic analysis of endocytosis and adaptor recruitment to show that μ2, a subunit of AP2 interacts directly with phospholipase D (PLD)1, a receptor-associated signaling protein and this facilitates the membrane recruitment of AP2 and the endocytosis of epidermal growth factor receptor (EGFR). We also demonstrate that the PLD1-μ2 interaction requires the binding of PLD1 with phosphatidic acid, its own product.

**Conclusions/Significance:**

These results suggest that the temporal regulation of EGFR endocytosis is achieved by auto-regulatory PLD1 which senses the receptor activation and triggers the translocation of AP2 near to the activated receptor.

## Introduction

The internalization of receptors is a complex process orchestrated by multiple proteins including clathrin, endocytic proteins, and adaptor proteins, which recruit their cargo into clathrin-coated pits (CCPs) [1], [2]. Heterotetrameric AP2, which contains α, β2, μ2, and σ2 subunits is a key adaptor in clathrin-mediated endocytosis (CME) [3]. It triggers clathrin assembly, recruits endocytic accessory proteins, and interacts directly with internalization motif of cargo molecules through its β2, α, and μ2 subunit respectively [4]. It has been generally accepted that AP2 complex is required for the endocytosis of cell surface receptors. However, it is still the subjects of debate how AP2 roles in the internalization of activated receptor [5], [6], [7] and what determines the kinetics of AP2 recruitment to the activated receptor and receptor endocytosis.

Upon EGF binding, EGFR is activated and internalized from the cell surface via clathrin coated pits by the action of endocytic proteins [8]. PLD1 is a receptor-associated signaling enzyme catalyzing the hydrolysis of phosphatidylcholine (PC) to choline and phosphatidic acid (PA) [9]. Although a previous study suggested that the lipase activity of PLD1 might be involved in EGFR endocytosis based on the overexpression strategy [10], direct evidence for the involvement of endogenous PLD1 lipase activity is lacking and the underlying mechanism is largely unknown.

In this study, we describe the role of PLD1 in the EGF stimulation-induced AP2 translocation and its involvement in the kinetic regulation of EGFR endocytosis. We propose that PLD1 roles as a membrane docking site for AP2 and that the functional downstream target of PLD1 lipase activity is PLD1 itself. Our findings provide novel insights into the unique working model of PLD1 as a signaling timer for EGFR internalization.

## Results

### Wild type but not lipase inactive PLD1 facilitates EGFR endocytosis

**Figure 1:**
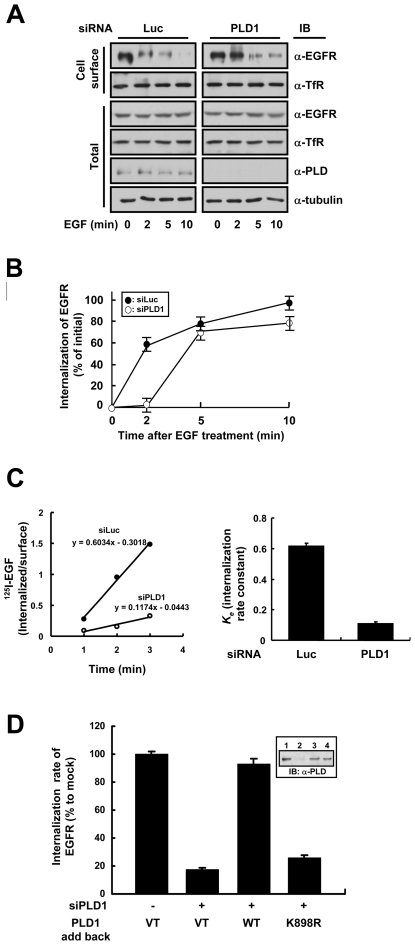
Wild type but not lipase inactive PLD1 facilitates EGFR endocytosis. (A) HeLa cells were transfected with control (Luc) or PLD1 (PLD1) siRNA to deplete endogenously expressed PLD1. After serum starvation for 12 h, EGF was treated at 20 nM for 0, 2, 5, or 10 min and biotinylation of cell surface proteins was performed. Biotinylated (Cell surface) proteins were separated using streptavidin beads and analyzed by western blotting. A representative immunoblot of three independent experiments is shown. (B) Quantitation of EGFR internalization in HeLa cells. The kinetics of the EGF-induced internalization of EGFR in HeLa cells transfected with either luciferase (closed circle) or PLD1 (open circle) siRNA. (C) HeLa cells depleted of endogenous PLD1 were incubated with ^125^I-EGF at 37°C. *k_e_* valueswere measured during linear 3-min time course and expressed as per cent of the mean values obtained for control-transfected cells. The data represent mean values from three independent experiments and the error bars represent standard deviations. (D) After depleting endogenous PLD1 using PLD1 siRNA, endogenous level of wild type or K898R PLD1 was expressed in HeLa cells. The internalization rate of EGFR was calculated as described in (C) and is shown as a bar graph reflecting the average of three independent experiments and standard deviations. Immunoblots in the *inset* indicate the expression levels of the constructs used.

To investigate the involvement of endogenous PLD1 in EGFR endocytosis, we designed siRNA for human PLD1 (siPLD1), corresponding to the human PLD1a coding nucleotides 1455–1475, and measured the internalization of EGFR in HeLa cells. The designed siPLD1 successfully reduced the endogenous expression of PLD1 to <10% of the control (i.e., inhibition with luciferase siRNA: siLuc) within 72 hours of transfection (data not shown). Cell surface protein biotinylation studies showed that the EGF (20 nM)-induced endocytosis of EGFR was significantly delayed in cells transfected with siPLD1 compared with the control (Figure 1A; see also Figure 1B for quantitative analysis). The maximal attenuation of EGFR internalization occurred after 2 min of EGF treatment. However, the internalization of transferrin receptor (TfR), which constitutively endocytoses through clathrin-coated pits, remained unchanged. The ^125^I-EFG internalization data strongly support the essential role of PLD1 in EGFR internalization (Figure 1C). The strong inhibitory effect of PLD1 depletion by siRNA on the uptake of was observed during linear 3-min time course. The internalization rate constant (*k_e_*) of PLD1-depleted cells was 19.5% compared to control. To confirm the role of PLD1 and to check the involvement of the lipase activity of PLD1 in EGFR endocytosis, we transfected wild type or a lipase inactive mutant PLD1 (K898R) after depleting the endogenous PLD1 from HeLa cells by siPLD1, and measured the internalization of EGFR after 2 min of EGF treatment. The expression of wild type PLD1 in PLD1-depleted HeLa cells restored the internalization rate of EGFR. However, the expression of PLD1 (K898R) failed to recover EGFR internalization rate (Figure 1D). These results suggest that endogenous PLD1 facilitates the EGF-induced internalization of EGFR in HeLa cells in a lipase activity-dependent manner.

### PLD1 associates with the μ2 subunit of AP2 in a PA-dependent manner

**Figure 2:**
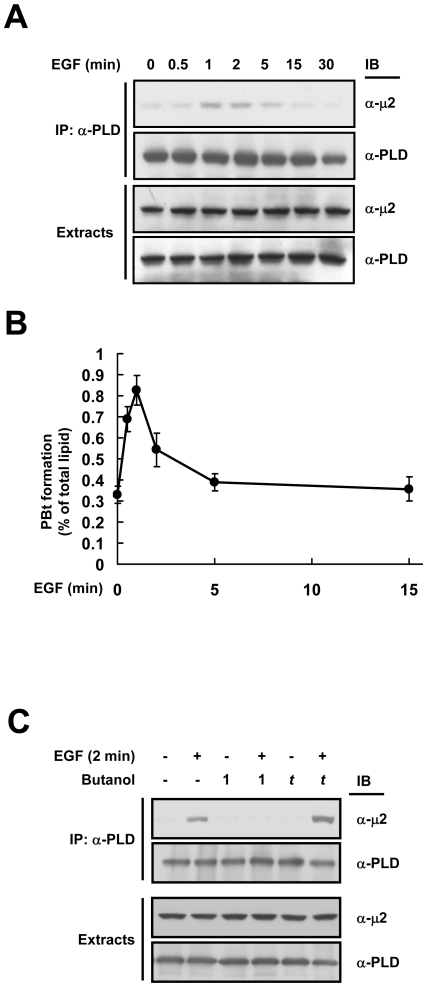
PLD1 associates with the μ2 subunit of AP2 in a PA-dependent manner. (A) HeLa cells were treated with EGF (20 nM) and cell extracts were immunoprecipitated with anti-PLD antibody and then immunoblotted with the indicated antibodies. (B) The kinetic of EGF-induced PLD1 activation in HeLa cells. (C) HeLa cells were treated with EGF (20 nM) for 1 min in the absence or presence of either 1-butanol (0.4%) or *t*-butanol (0.4%) as shown and then the cell lysates were immunoprecipitated as in (A). Alcohols were added 5 min prior to EGF treatment.

The endocytosis of activated cell surface receptors is mediated by an adaptor protein AP2 that recognizes them via its μ2 subunit and recruits dynamin, clathrin, and accessory proteins involved in vesicle formation [4]. PLD1 localizes near EGFR in the plasma membrane and is transiently activated upon EGF stimulation [11]. To understand the mechanism by which lipase activity of PLD1 facilitates EGFR endocytosis, we checked the possibility of EGF stimulation-dependent complex formation between PLD1 and the components of AP2. Interestingly, the μ2 subunit of AP2 was found to transiently interact with endogenous PLD1 after 1–2 min of EGF stimulation in HeLa cells (Figure 2A). In contrast, the lipase inactive mutant PLD1 (K898R), which could not enhance EGFR endocytosis (see Figure 1C) failed to interact with μ2 (data not shown). To investigate the involvement of the lipase activity of PLD1 in its binding to μ2, we measured the kinetics of PLD1 activation under the same conditions after EGF treatment. Figure 2B shows that the activity of PLD1 dramatically stimulated after 1 min of EGF treatment which correlates well with the kinetics of PLD1-μ2 interaction. To confirm the lipase activity-mediated interaction between PLD1 and μ2, 1-butanol was used to inhibit the PA production. PLD preferentially utilizes primary alcohol (e.g., 1-butanol), but not tertiary alcohol (e.g., *t*-butanol), over water to generate a phosphatidylalcohol (e.g., phosphatidylbutanol) instead of PA [12]. After 2 min of EGF stimulation, the association between PLD1 and μ2 was detected in control and *t*-butanol treated cells, but not in 1-butanol treated cells (Figure 2C). Taken together, these results suggest that the production of PA by PLD1 is important for its interaction with μ2.

### Association with PA enhances the direct interaction between PLD1 and μ2

**Figure 3:**
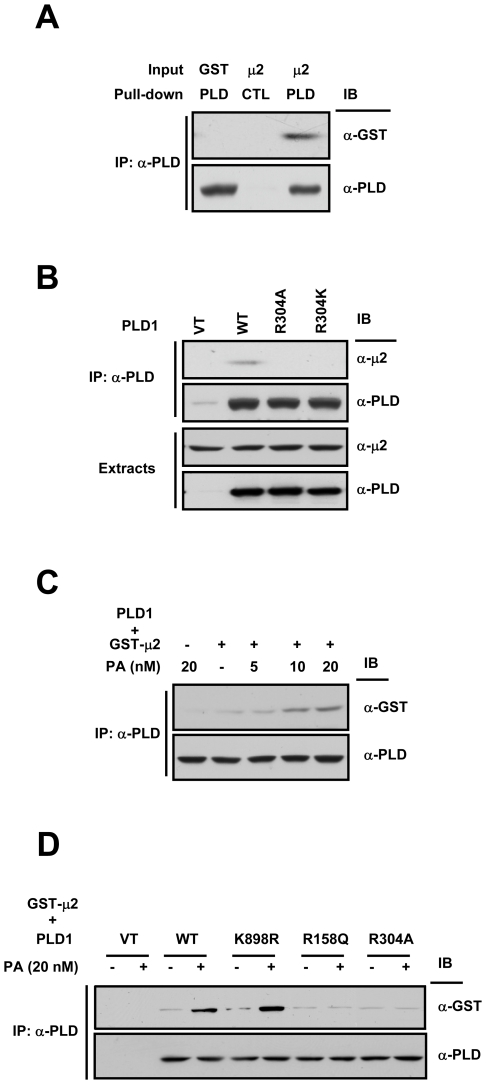
Association with PA enhances the direct interaction between PLD1 and μ2. (A) Purified PLD1 immobilized with anti-PLD antibody (PLD) or with pre-immune serum (CTL) was incubated with purified GST alone or GST-μ2. (B) HeLa cells expressing vector, wild type, R304A, or R304K PLD1 were grown in DMEM containing 10% fetal bovine serum. Cells were lysed and immunoprecipitated with anti-PLD antibody. (C) Purified PLD1 immobilized with anti-PLD antibody was incubated with purified GST alone or GST-μ2 in the absence or presence of C6-PA at the indicated concentrations. (D) Vector, wild type, K898R, or R158Q PLD1 transiently expressed in HeLa cells was immunoprecipitated and incubated with purified GST-μ2 in the absence or presence of 20 nM of C6-PA.

To test whether PLD1 directly interacts with μ2 or not, *in vitro* pull-down analysis was performed using purified PLD1 and GST-μ2. As shown in Figure 3A, GST-μ2 was coprecipitated with PLD1, indicating that μ2 binds directly to PLD1. PLD1 is composed of a phox homology (PX) domain, a pleckstrin homology (PH) domain, a central loop, and the conserved region (CR) I-IV [13]. To identify the region responsible for direct binding to μ2, we used GST fusion PLD1 fragments as shown schematically in Figure S1A. The pull-down assay showed that the PH domain bound to μ2 (Figure S1B and S1C) via a region spanning amino acids 296–312 (Figure S1D). Moreover, this μ2-PLD1 PH domain interaction was highly specific, as the PH domains of other proteins did not show any binding to μ2 (Figure S1E). When immunoprecipitation was performed in HeLa cells that were transfected with PLD1 constructs after depleting endogenous PLD1, μ2 bound to wild type PLD1, but not to R304A or R304K mutant (Figure 3B). These results indicate that Arg 304 within the PH domain of PLD1 is directly involved in interaction with μ2.

We then investigated the mechanism whereby PA induces the interaction between PLD1 and μ2. Stahelin et al. [14] suggested that the PX domain of PLD1 has a binding pocket for anionic phospholipids such as PA. Thus, we hypothesized that the occupation of the PX domain by PA generated by the activation of PLD1 might contribute to the binding of the PH domain and μ2. To explore this possibility, we checked the effect of PA on the PLD1-μ2 interaction using exogenous PA, and found that PA increased this interaction in a dose-dependent manner (Figure 3C). Furthermore, the addition of 20 nM of PA induced binding between μ2 and the lipase inactive mutant PLD1 (K898R), as shown in Figure 3D. Interestingly, PLD1 (R158Q) mutant, which has full lipase activity (data not shown) but reduced affinity for PA [14], could not interact with μ2 in the presence of 20 nM of PA. These results indicate that PLD1 is a direct target of PA, its own product, and that the PA binding enhances its interaction with μ2.

### The PLD1-μ2 interaction is important for adaptor recruitment onto membranes

**Figure 4:**
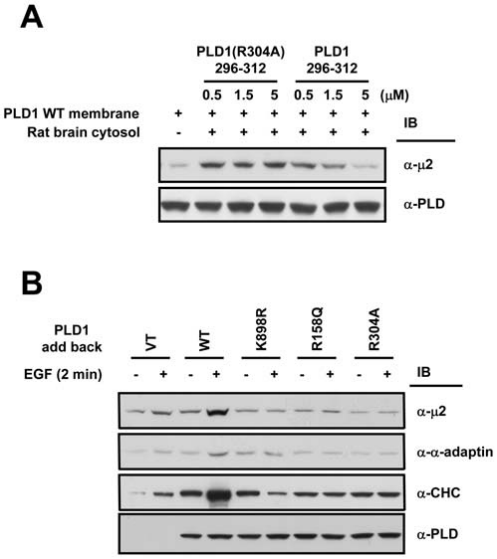
The PLD1-μ2 interaction is important for adaptor recruitment onto membranes. (A) Purified GST-fusion PLD1 296–312 or PLD1 296–312 (R304A) was added to recruitment assay mixture containing membrane preparation from wild type PLD1-expressing HeLa cells and rat brain cytosol (10 mg/ml). Membrane-recruited μ2 was detected by western blotting. Data are representative of two independent experiments. (B) Translocation of endocytic proteins onto plasma membrane was analyzed. HeLa cells transfected with vector, wild type, K898R, R158Q, or R304A PLD1 were treated with EGF (20 nM) during 2 min and the membrane preparations were subjected to SDS-PAGE and immunoblotted using the indicated antibodies.

AP2 localizes to the cytosol when cells are in an un-stimulated state, thus AP2 should translocate onto the plasma membrane before it acts as an adaptor linking EGFR to the endocytic machinery [15]. Also, it is generally accepted that recognition of EGFR by μ2 is an essential process in the clathrin-mediated endocytosis of EGFR [4]. To understand the role of PLD1-μ2 interaction in EGFR endocytosis, we first performed in vitro membrane recruitment analysis using membrane preparation of wild type PLD1 and rat brain cytosol. The addition of purified PLD1 fragment (296–312) corresponding to the region involved in μ2 binding abolished the positive effect of wild type PLD1 on the recruitment of μ2 onto the membrane in a dose-dependent manner (Figure 4A); however, the same fragment carrying the R304A mutation had no effect. To confirm this, we performed adaptor recruitment analysis in cells. HeLa cells depleted of endogenous PLD1 were transfected with wild type, K898R, R158Q, or R304A PLD1 and the localization of endocytic proteins were monitored after 2 min of EGF treatment. Wild type PLD1, but none of the mutants with little affinity for μ2 (i.e., K898R, R158Q, and R304A), enhanced the recruitment of μ2, α-subunit of AP2 (α-adaptin), and clathrin heavy chain (CHC) onto membrane (Figure 4B). These results suggest that the interaction between PLD1 and its reaction product PA promotes the recruitment of adaptors to membranes by enhancing the interaction between PLD1 and μ2.

### PLD1-μ2 interaction is important for PLD1-mediated EGFR endocytosis

**Figure 5:**
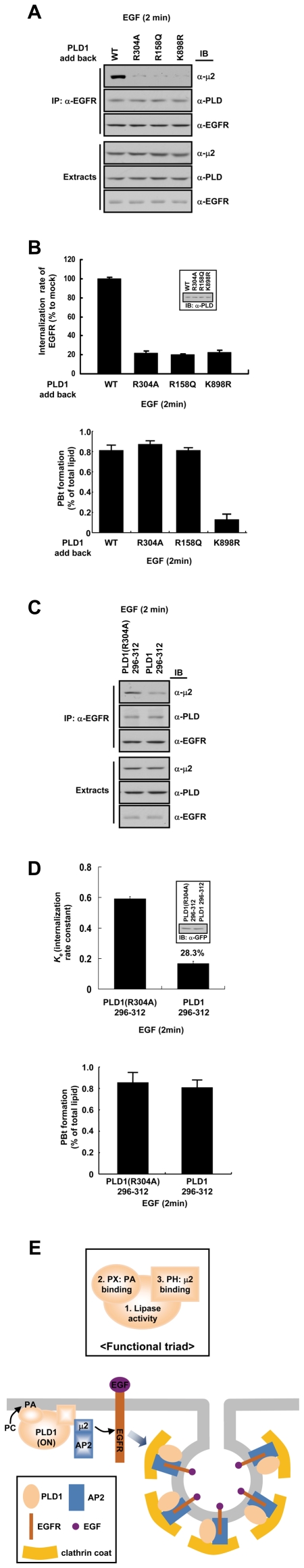
PLD1-μ2 interaction is important for PLD1-mediated EGFR endocytosis. (A) The recognition of EGFR by μ2 was analyzed by western blotting. Cell extracts were immunoprecipitated with anti-EGFR antibody and then immunoblotted using the indicated antibodies. (B) HeLa cells were transfected with wild type, R304A, R158Q, or K898R PLD1 after being depleted of endogenous PLD1. After 2 min of EGF (20 nM) treatment, the internalization rate of EGFR was measured as in Figure 1 (D). The PLD1 lipase activity was measured in a parallel experiment. Immunoblot in the *insets* indicates the expression levels of the constructs used. (C) and (D) The effect of PLD1-μ2 interaction on EGFR-μ2 association (C) and EGFR internalization rate (D) was checked. (E) Schematic representation of the proposed mechanism whereby PLD1 facilitates EGFR endocytosis in an auto-regulatory manner. Upon EGF stimulation, PLD1 is activated and generates PA. Association between PLD1 and PA through the PX domain of PLD1 increases the affinity of the interaction between the PH domain of PLD1 and μ2. This interaction enhances the translocation of AP2 onto the plasma membrane and the recognition of EGFR by μ2 facilitating EGFR endocytosis.

Upon EGF stimulation, μ2 translocates onto the plasma membrane and recognizes EGFR. So we checked the role of PLD1 in the μ2-EGFR interaction. EGF-dependent EGFR-μ2 association was observed in wild type PLD1-transfected cells, but not in cells transfected with PLD1 (R304A), PLD1 (R158Q), or PLD1 (K898R) although these PLD1 constructs exist in EGFR complex (Figure 5A). Next, we measured the internalization rate of EGFR during 3 min of EGF treatment. Cells expressing PLD1 (R304A), PLD1 (R158Q), or PLD1 (K898R) neither of which could interact with μ2 showed about 20% of EGFR internalization rate compared to wild type PLD1-expressing cells (Figure 5B). These PLD1 constructs were expressed in comparable levels in cells (Figure 5B, inset) and had comparable lipase activity. In addition, the transient expression of a PLD1 fragment 296–312 resulted in the potent inhibition of EGFR internalization rate (Figure 5D) and the EGFR-μ2 association (Figure 5C) although this construct had little effect on the lipase activity of PLD. Taken together, these results indicate that the interaction between PLD1 and μ2 is a key determinant for the EGF-induced internalization rate of EGFR in cells.

## Discussion

PLD1 is a key factor in the regulation of EGFR endocytosis. PLD1 is activated upon EGF stimulation and it hydrolyzes PC to PA at the plasma membrane [16]. Nonetheless, the identity of PA effector protein and the mechanism by which PA regulates the EGFR endocytosis are still unknown. The present study shows that PLD1 itself is an effector of PA and auto-regulatory interaction between the PX domain of PLD1 and PA promotes the binding of another domain of PLD1, the PH domain, with μ2 and thereby facilitates EGFR endocytosis. The findings that the PLD1-μ2 association is dependent on its lipase activity and that the effector of PA is PLD1 itself establish the key role of PLD1 in EGFR endocytosis and also provide new mechanistic insight. To our knowledge, this is the first example of an auto-regulatory mechanism for a signaling enzyme.

On the basis of our results, we propose that PLD1 is a complex molecular device with functional triad composed of catalytic, PX, and PH domain, each of which senses and mediates discrete steps of EGFR endocytosis (Figure 5E). In our model, the catalytic domain of PLD1 first generates PA from PC in response to EGFR activation. The PX domain of PLD1 then binds with PA and thus senses the input indicating that EGFR has been activated. This in turn may cause the conformational change of PLD1 to expose the μ2 binding site of the PH domain which generates the output signal that triggers the translocation of AP2 to the plasma membrane and the endocytosis of EGFR. Previously, we also reported that the PX domain of PLD1 has GTPase activating properties for dynamin and enhances the EGFR endocytosis at lower concentration of EGF [17]. Therefore, PLD1 might be a multifunctional hub protein for the control of EGFR endocytosis speed in response to the strength of the outside EGF signal.

It is still unclear how the kinetics of receptor endocytosis is finely regulated depending on the strength of receptor activation. In this study, we found direct evidence linking receptor stimulation and endocytic molecule recruitment. We observed that increased concentration of EGF (20 nM)-induced PLD1 activation is responsible for the translocation of endocytic adaptor protein (Figure 4B) and that this is critically required to facilitate the internalization of EGF receptor (Figure 5B. When EGF is present at low concentrations, EGFR endocytosis occurs slowly (linear increase, time for half internalization (t_1/2_) is about 5 min) and under such conditions the GAP function of PLD1, which is independent of its lipase activity, rather than its lipase activity is mainly involved, because the amount of EGF is not enough to induce PLD1 lipase activity. However, when EGF is present at relatively high concentrations to turn on PLD activity, PLD1 performs an additional role in endocytosis (in addition to acting as a GAP for dynamin) by acting as a membrane target for the adaptor protein, which enables for a high-rate of internalization of EGFR (highly saturated, t_1/2_≈1.5 min). PLD2, an isoform of PLD1, also roles in EGFR endocytosis by acting as GAP for dynamin [17] (lipase activity-independent role) but not in lipase activity-dependent manner because it does not interact with μ2 (data not shown). These results imply that receptor endocytosis utilizes the same molecules but has a regulatory mode that depends on the amount of ligand.

The present study demonstrates that PA generated by PLD1 activation plays a pivotal role in the facilitated EGFR endocytosis. PA roles in promoting the PLD1-μ2 interaction and triggering the membrane translocation of AP2 (see Figure 4B) through binding to the PX domain of PLD1. Furthermore, a good correlation between the activation kinetics of PLD1 and the kinetics of PLD1-μ2 interaction and EGFR endocytosis (see Figure 2B, 2A, and 1A respectively) suggests that PLD1 controls the kinetics of EGFR endocytosis through the generation of PA. It has been proposed that PA stimulates PIP5-kinase which is primarily associated with the plasma membrane [18] and generates phosphatidylinositol–4,5-bisphosphate (PIP_2_) [19] which plays a pivotal role in receptor endocytosis. The level of PIP_2_ reaches a maximum at the initiation of endocytosis [20] and it causes the recruitment of endocytic proteins onto the plasma membrane [21], [22]. Recently it was also reported that activation of PIP5-kinase through interaction with AP2 and local generation of PIP_2_ is important for the recruitment of endocytic machineries and synaptic vesicle internalization [23]. Taken together, we propose that PA generation by PLD1 activation is a key molecular event that causes the local accumulation of PIP_2_ through direct activation of PIP5-kinase and enhancing AP2-PIP5-kinase pathway by placing these two molecules in close proximity to facilitate EGFR endocytosis.

## Materials and Methods

### RNA interference

Pairs of 21-nucleotide sense and antisense RNA oligomers which correspond to human PLD1a coding nucleotides 1455–1475 were chemically synthesized and annealed by Dharmacon Research, Inc. The oligonucleotides for PLD1 were as follows: sense, 5′-AAG GUG GGA CGA CAA UGA GCA-3′, and antisense, 5′-UGC UCA UUG UCG UCC CAC CUU-3′. Luciferase GL2 duplex (Dharmacon Research, Inc.) was used as negative control.

### Cell culture and transfection

HeLa cells obtained from the American Type Culture Collection were cultured in Dulbecco's modified Eagle's medium (DMEM) containing 100 units/ml penicillin, 100 µg/ml streptomycin, and 10% fetal bovine serum (GIBCO-BRL). HeLa cells were introduced with synthetic siRNA (20 nM duplex) for PLD1 using METAFECTENE™ reagent (Biontex) according to the manufacturer's instructions and then cultured for 48 hours to achieve PLD1 silencing. To express the PLD1 constructs, cells were transiently expressed with rat-origin PLD1 cDNAs using Lipofectamine Plus™ reagent (Invitrogen) one day after siRNA transfection.

### Generation of plasmids and GST fusion proteins

R304A and R304K point mutants of PLD1 were generated by mutating the arginine residue at 304 to Ala or Lys respectively. Briefly, the R304A mutant was obtained using forward primer: 5′-TGA TAA TCT TTC AGC TAC ACT GAT TTT A-3′ and reverse primer: 5′-TAA AAT CAG TGT AGC TGA AAG ATT ATC A-3′. A similar strategy was used to generate the R304K mutant using forward primer: 5′-TGA TAA TCT TTC AAA AAC ACT GAT TTT A-3′ and reverse primer: 5′-TAA AAT CAG TGT TTT TGA AAG ATT ATC A-3′. The resulting products were cloned into pCDNA3.1 (Invitrogen) or pEGFP-C1 (Clontech) vector. Glutathione *S*-transferase (GST) fusion proteins were prepared using glutathione-Sepharose 4B (Amersham Biosciences). GST fusion PH domains of PLCβ1, IRS, and dynamin were kindly provided by Dr. Brian K. Kay (The University of Wisconsin, Madison, WI) and GST-μ2 was generous gift from Dr. Juan S. Bonifacino (National Institutes of Health, Bethesda, MD).

### *In vitro* binding analysis

*In vitro* binding was performed in 500 µl of buffer A (50 mM Hepes-NaOH (pH 7.5), 3 mM MgCl_2_, 2 mM CaCl_2_, 3 mM EGTA, and 80 mM KCl) containing 1% Triton X-100 at 4°C for 3 h. In experiments adding C6-PA (Avanti Polar Lipids), pellets were washed with buffer A containing the same concentrations of C6-PA used in the incubation.

### Immunoprecipitation and western blotting

For immunoprecipitation, HeLa cells were serum starved for 12 h, treated with EGF (20 nM) (Daewoong Pharmaceutical Co.), and lysed by sonication with buffer A containing protease inhibitors, 1% Triton X-100, and 1% cholic acid. Cell lysates were centrifuged at 100,000 *g* for 30 min at 4°C and the same amounts of supernatant were incubated with anti-PLD [24] or anti-EGFR (Upstate Biotechnology) antibody immobilized on Protein A-Sepharose beads (Amersham Biosciences). For immunoblotting, anti-μ2 (BD Transduction Laboratories), anti-α-adaptin (BD Transduction Laboratories), anti-clathrin heavy chain (BD Pharmingen), and anti-GST (Santa Cruz Biotechnology, Inc.) antibodies were used. Horseradish peroxidase-conjugated goat anti-rabbit or goat anti-mouse antibodies were purchased from Kirkegaard and Perry Laboratories Inc. Proteins were visualized by chemiluminescence using Enhanced Chemiluminescence kit (Amersham Bioscience).

### *In vitro* adaptor recruitment assays

HeLa cells transfected with wild type PLD1 were homogenized with buffer B (buffer A with protease inhibitors and 0.25 M sucrose) using a Dounce homogenizer and then sonicated. A postmitochondrial supernatant was prepared by centrifuging the homogenate at 1500 *g* for 10 min and the resulting supernatant was further centrifuged at 100,000 *g* for 15 min in a TL-100.2 rotor (Beckman Instruments). After washing with buffer B, membrane pellets were resuspended in 50 µl of rat brain cytosol (10 mg/ml) prepared in buffer A containing protease inhibitors. After 15 min at 37°C, reactions were stopped by adding 1 ml of cold buffer A and membranes were collected by centrifugation as described above. Pellets were resuspended in 50 µl of Laemmli sample buffer and 10 µl aliquots were subjected to SDS-PAGE and western blotting.

### Adaptor recruitment assays in cells

Recruitment of endocytic proteins onto cell membranes was performed as described below. After depletion of endogenous PLD1, HeLa cells were transfected with vector, wild type, K898R, R158Q, or R304A PLD1. The cells were then treated with EGF (20 nM) during 2 min and washed twice with ice-cold buffer A. Preparation of cell membrane was performed as described in “*In vitro* adaptor recruitment assays.” After washing with buffer B, membrane pellets were resuspended in 50 µl of Laemmli sample buffer and 10 µl aliquots were subjected to SDS-PAGE and western blotting.

### Internalization of EGFR

HeLa cells grown in 60 mm dishes were starved for 12 h and EGF (20 nM) was treated. Cells were washed twice with ice-cold PBS containing 0.1 mM CaCl_2_ and 1 mM MgCl_2_ (PBS^2+^) and incubated with 1 mg/ml Sulfo-NHS-SS-biotin (Pierce) for 30 min at 4°C. Nonreactive biotin was quenched with ice-cold PBS^2+^ and 0.1 M glycine for 20 min at 4°C. The cells were then lysed with radioimmunoprecipitation assay (RIPA) buffer (10 mM Tris (pH 7.4), 150 mM NaCl, 1 mM EDTA, 0.1% SDS, 1% Triton X-100, and 1% sodium deoxycholate) containing protease inhibitors and protein concentrations were determined. Biotinylated and nonbiotinylated proteins were separated from equal amounts of cellular protein by incubation with Immunopure immobilized streptavidin (Pierce) for 4 h at 4°C. The biotin-streptavidin-agarose complexes were harvested by centrifugation and washed five times with RIPA buffer. Proteins bound to beads were eluted in Laemmli sample buffer and analyzed by SDS-PAGE and western blotting. The ratio of internalized EGFR was determined by densitometric analysis.

### Internalization of ^125^I-EGF

HeLa cells grown in 12-well dishes were treated with ^125^I-EGF at 37°C and the ratio of internalized and surface radioactivity was determined during linear 3-min to calculate the specific internalization rate constant *k_e_* as described previously [7].

### *In vivo* PLD activity assays

PLD activity analysis was performed as previously described [25]. After 24 h of fasting, the HeLa cells were incubated with 10 µCi [^3^H]myristic acid (PerkinElmer Life Sciences) for 3 h. PLD activity was determined using the transphosphatidylation reaction in the presence of 0.4% 1-butanol. Lipids were extracted and separated by Silica Gel 60 thin-layer chromatography (chloroform∶ methanol∶ acetic acid, 90∶10∶10 volume ratio), and the amounts of labeled phosphatidylbutanol and total lipid were determined using a Fuji BAS-2000 image analyzer (Fuji Film).

## Supporting Information

Figure S1 Binding region mapping of PLD1 for μ2. (A) Schematic depictions of PLD1 fragments. (B–E) The interaction between PLD1 and μ2 was analyzed by pull-down assay and western blotting. The same amounts of GST alone or GST fusion PLD1 fragments (B), PLD1 PX domain and the PH domain (C), fragments of the PLD1 PH domain (D), and PH domains from PLD1, PLCβ-1, IRS-1, and dynamin-1 (E) were incubated with purified μ2.
(0.73 MB TIF)
