# Supplementary figures and images for: Determination of EGFR Endocytosis Kinetic by Auto-Regulatory Association of PLD1 with μ2
Source: PLoS One. 2009 Sep 18;4(9):e7090. doi: 10.1371/journal.pone.0007090 (PMC2739277; doi:10.1371/journal.pone.0007090)

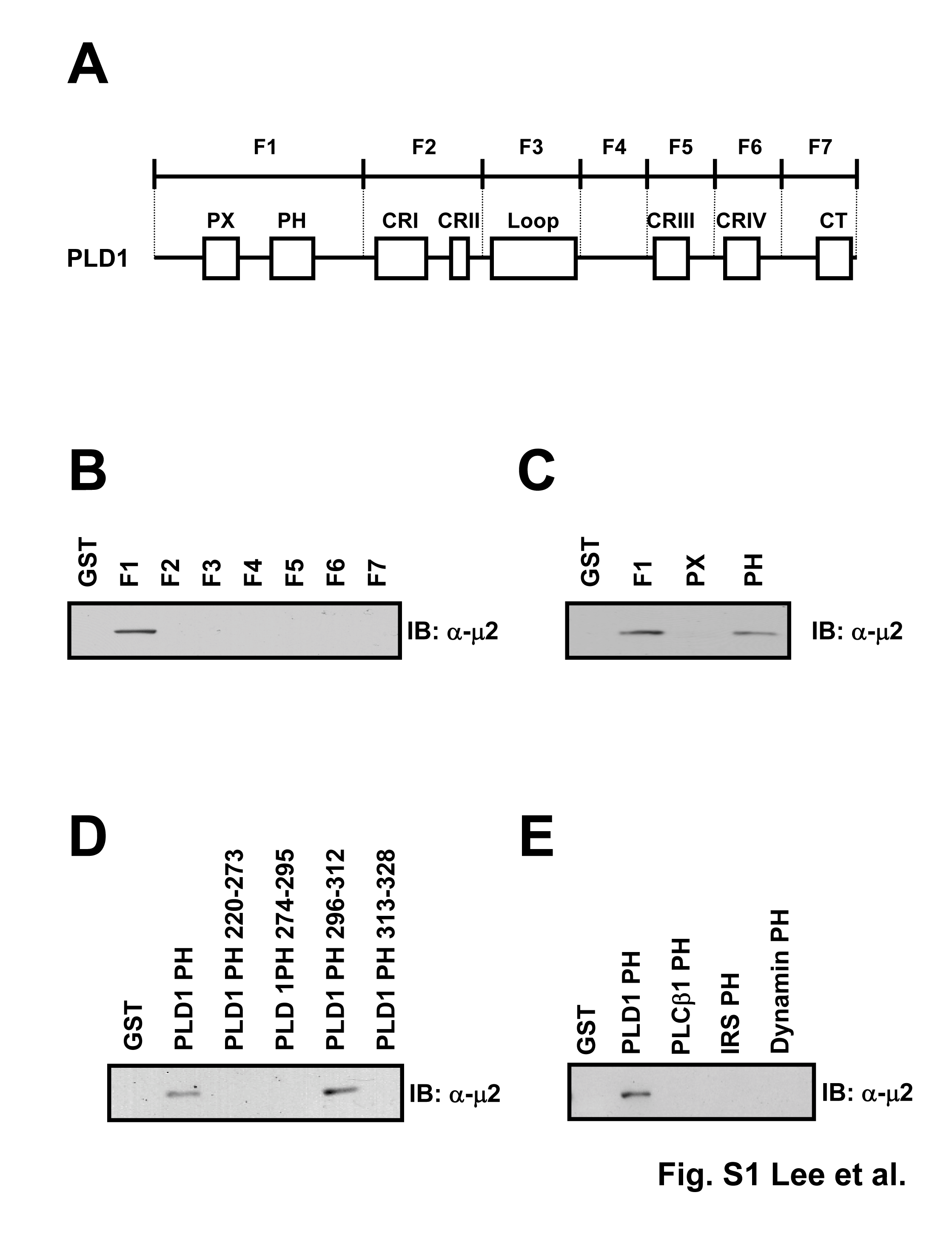

Supplement: Figure S1 — Binding region mapping of PLD1 for μ2. (A) Schematic depictions of PLD1 fragments. (B–E) The interaction between PLD1 and μ2 was analyzed by pull-down assay and western blotting. The same amounts of GST alone or GST fusion PLD1 fragments (B), PLD1 PX domain and the PH domain (C), fragments of the PLD1 PH domain (D), and PH domains from PLD1, PLCβ-1, IRS-1, and dynamin-1 (E) were incubated with purified μ2. (0.73 MB TIF) [file pone.0007090.s001.tif]
